# The effect of chemotherapy in patients with stage I mucinous ovarian cancer undergoing fertility-sparing surgery

**DOI:** 10.3389/fonc.2022.1028842

**Published:** 2022-11-08

**Authors:** Xingtao Long, Rengui Li, Ying Tang, Lingling Yang, Dongling Zou

**Affiliations:** Gynecological Oncology Center, Chongqing University Cancer Hospital, Chongqing, China

**Keywords:** SEER, mucinous ovarian cancer, fertility preservation, chemotherapy, prognosis

## Abstract

**Objective:**

To determine the effect of adjuvant chemotherapy in patients with stage I mucinous ovarian cancer (MOC) undergoing fertility-preserving surgery.

**Patients and methods:**

The clinicopathological characteristics and survival information of young women with stage I MOC from SEER databases between 2004 and 2019 were collected. The relationship between chemotherapy and the characteristics was examined by univariate and multivariate logistic regression analyses. Univariable and multivariate Cox proportional hazards survival analysis were employed for cancer-specific survival. Cox analysis was performed to build a nomogram model.

**Results:**

All 901 eligible patients with stage I MOC were screened from the SEER database. There were 321(35.6%) patients aged 9-30 years, 580(64.4%) aged 31-45 years, 645 (71.6%) patients with stage IA/IB, 256 (28.4%) with stage IC disease, 411(45.6%) who underwent fertility-sparing surgery, and276(30.6%) who received postoperative adjuvant chemotherapy. Multivariate logistic regression analyses showed that postoperative chemotherapy was often used in patients aged 31-45 relative to aged 9-30 (HR: 2.215, 95%CI 1.443-3.401, P < 0.001) or with grade 3 compared to grade 1 tumors (HR: 7.382, 95%CI 4.054-13.443, P < 0.001) or with stage IC compared to stage IA/IB (HR: 6.436, 95%CI 4.515-9.175, P < 0.001) or with non-fertility sparing surgery relative to fertility-sparing (HR:2.226, 95%CI 1.490-3.327, P < 0.001). Multivariate analysis for the special population with fertility preservation indicated that patients with chemotherapy (HR: 2.905, 95% CI: 0.938-6.030, P=0.068) or with grade 3 (HR: 4.750, 95% CI: 1.419-15.896, P=0.011) had a greater risk of mortality. Significant CSS differences were observed between the non-chemotherapy and chemotherapy groups in MOC when patients were stage IA/IB-grade 2 (P=0.004) (10-year CSS rates of chemotherapy=84%, non-chemotherapy = 100%), but not when they were stage IA/IB-grade 1, stage IA/IB-grade 3 or stage IC (both P>0.05). A prognostic prediction nomogram model was built for stage I MOC patient who underwent fertility-sparing and the C-index was 0.709.

**Discussion:**

The patients aged 31-45 years, with grade 3, stage IC, and non-fertility-sparing surgery were more likely to receive adjuvant chemotherapy in the real world. For stage I MOC patient who underwent fertility-sparing surgery, the choice of chemotherapy may increase the risk of death, and it should be carefully selected in clinical practice.

## Introduction

As reported, ovarian cancer is the second most common gynecological malignancy in terms of incidence, but it has the highest fatality rate of all gynecological tumors, which pose a serious threat to women’s health (1). It is well known that epithelial ovarian cancer, which includes serous cancer, mucinous cancer, clear cell cancer, endometrioid cancer, and other types, is the most frequent histological form of ovarian cancer. The biological and epidemiological characteristics of these different types of epithelial tumors are quite different which makes the treatment of ovarian cancer difficult (2, 3). According to reports (4), there are about 200,000 new cases of ovarian cancer worldwide each year (5). Mucinous ovarian cancer (MOC) is a rare histological subtype of epithelial ovarian cancer that makes up 3% of all cases (6). No clinical trials for MOC have been performed to date due to the low frequency, and patients with advanced MOC patients have a poor prognosis, which may be due to a poor response to platinum chemotherapy (7, 8). Drug resistance is linked to poor prognosis after recurrence (8). Mucinous ovarian cancer is more common in young females, and about 80% of patients are stage I when they were diagnosed (9). 5-year and 10-year survival rates was extremely high for stage I mucinous neoplasms but it remains controversial whether MOC patients need chemotherapy (10).

Hysterectomy and bilateral salpingo-oophorectomy are clinically recommended treatments for MOC, but for young female patients, this treatment means loss of fertility (11). The American Society of Clinical Oncology (ASCO) advised that the fertility of young women should be preserved as much as possible during treatment. For example, younger patients suffering from endometrial cancer and who undergo hysteroscopic endometrial resection or not, can be treated with oral progestins to maintain fertility (12). An increasing number of young people are opting for treatment that includes removal of tumor lesions while preserving fertility (13, 14). With the development of assisted reproductive technology, the fertility-sparing surgery is no longer limited to the preservation of the uterus and ovary, such as bilateral (salpingo-) oophorectomy without hysterectomy for patients with stage IB (15). However, it remains controversial whether patients with MOC need further chemotherapy after fertility-preserving treatment (16, 17). Chemotherapy may impair fertility in these young patients. The effect of surgery and chemotherapy in stage I mucinous ovarian cancer patients is still unknown (18, 19).

The Surveillance, Epidemiology, and End Results (SEER) database, which includes extensive real information of patients diagnosed with cancer from the United States, is one of the largest and most comprehensively available cancer database for the public. Here, we aimed to find the impact of chemotherapy and non-chemotherapy on the special population of fertility preservation by screening the SEER database.

## Methods

### Data source

**Figure 1:**
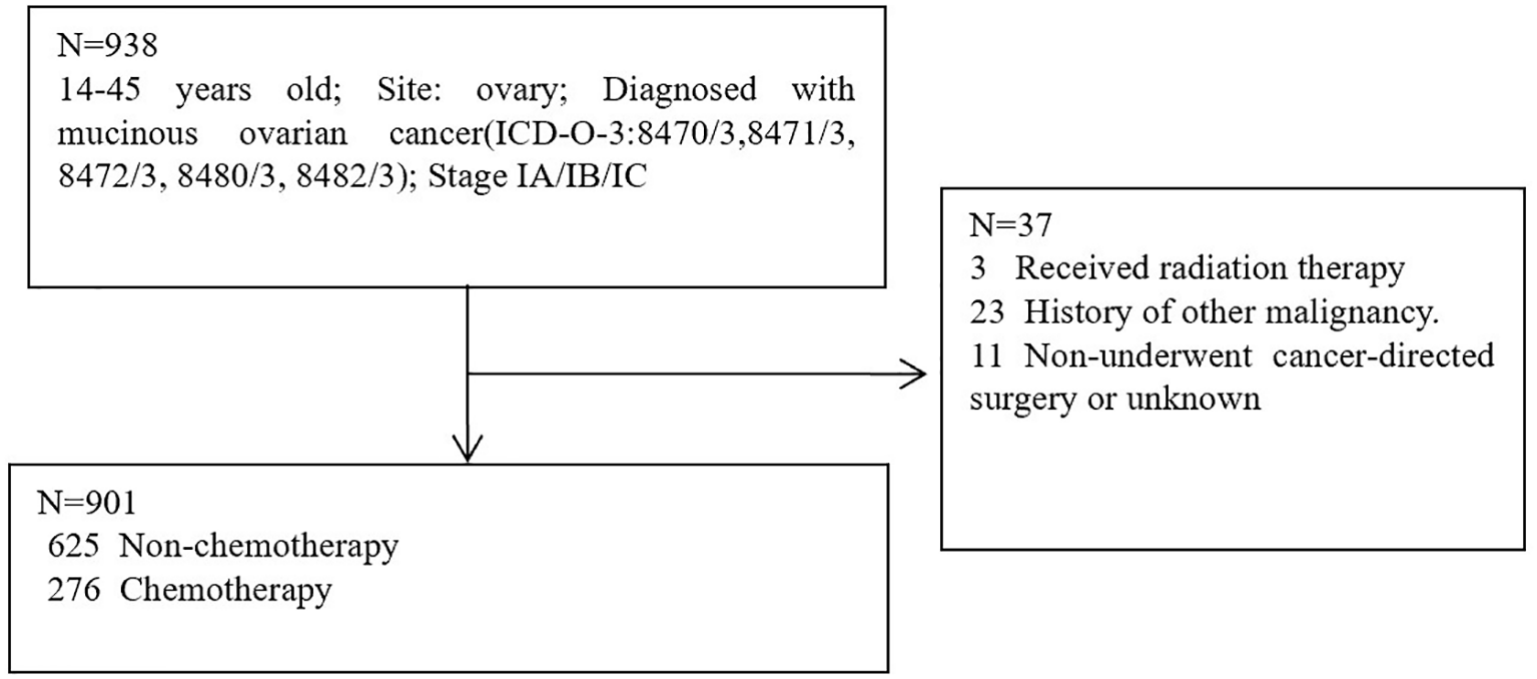
Flowchart of screening data from the SEER database.

The patient data were screened for this research from the SEER database. SEER∗Stat version 8.4.0.1 was used to obtain eligible data. Patients with histologically confirmed primary MOC between 2004 and 2019 were screened from the SEER database. SEER database: Incidence-SEER Plus Date, 8 Registries, Nov 2021 sub (1975–2019); Incidence-SEER Plus Date, 12 Registries, Nov 2021 sub (1992-2019); Incidence-SEER Plus Date, 17 Registries, Nov 2021 sub (2000-2019). Inclusion Criteria: 9-45 years old; Site: ovary; Diagnosed with mucinous ovarian cancer (ICD-O-3:8470/3,8471/3, 8472/3, 8480/3, 8482/3); Stage IA/IB/IC. Exclusion criteria: Received radiation therapy; History of other malignancy; Non-underwent cancer-directed surgery or unknown. A total of 938 patients were eligible for inclusion Criteria, 37 patients were eligible for exclusion criteria, 901 patients were utilized for this study (**Figure 1**). Cancer-specific survival (CSS) was regarded as the primary study endpoint and the definition of CSS was the interval between the time of diagnosis and the time of MOC-related death. Definition of fertility-sparing surgery: Unilateral (salpingo-) oophorectomy without hysterectomy; Resection of ovary (wedge, subtotal, or partial) only without hysterectomy; Unilateral or bilateral (salpingo-) oophorectomy without hysterectomy; Bilateral (salpingo-) oophorectomy without hysterectomy for patients with stage IB.

### Statistical analysis

To identify clinical traits and associated variables in women with fertility preservation following chemotherapy, univariate and multivariate logistic regression was used and all variables were clinically important and significant enough and we need to take all these factors into account when developing chemotherapy regimens for our patients. From the logistic regression, odds ratios (OR) and 95% confidence intervals (CI) were obtained. The categorical data were examined using the chi-square test. COX Hazard Regression Analysis was performed for both univariate and multivariate analyses to assess the risk factors for patients. Forward modeling for multivariate analysis employed stepwise regression. Through the results of the Cox hazard regression analysis, the nomogram was created. Software such as SPSS (version 24.0; IBM Corporation, St. Louis, Missouri, USA) and R software (version.3.6.2; The R Project for Statistical Computing, TX, USA; http://www.r-project.org)were used to perform statistical analysis. The difference was statistically significant when P value<0.05. Calibration was run to calculate the consistency compared with the genuine outcome for nomogram. Survival analysis comparisons were performed using Kaplan-Meier plots and log-rank tests.

## Results

### Characteristics of patients

The basic information and clinical characteristics of all 901 patients are shown in **Table 1**.

**Table 1:** Summary of demographic, surgical pathologic, and treatment information for stage I MOC.

	All patients	Non-chemotherapy	Chemotherapy
Characteristic	N = 901	N =625	N = 276
	(%)	(%)	(%)
**Age (years)**			
9-30	321 (35.6)	213 (34.1)	108 (39.1)
31-45	580 (64.4)	412 (65.9)	168 (60.9)
**Race**			
White	674 (74.8)	476 (76.2)	198 (71.7)
Black	51 (5.7)	30 (4.8)	21 (7.6)
Other/Unknown	176 (19.5)	119 (19.0)	57 (20.7)
**Marital status**			
Married	381 (42.3)	273 (43.7)	108 (39.1)
Single (never married)	408 (45.3)	279 (44.6)	129 (46.8)
Other/Unknown	112 (12.4)	73 (11.7)	39 (14.1)
**CA125**			
Negative/normal	170 (18.9)	123 (19.7)	47 (17.0)
Positive/elevated	239 (26.5)	144 (23.0)	95 (34.4)
Other/Unknown	492 (54.6)	358 (57.3)	134 (48.6)
**Stage**			
IA/IB	645 (71.6)	517 (82.7)	128 (46.4)
IC	256 (28.4)	108 (17.3)	148 (53.6)
**Grade**			
1	349 (38.7)	280 (44.8)	69 (25.0)
2	295 (32.8)	166 (26.5)	129 (46.7)
3	74 (8.2)	31 (5.0)	43 (15.6)
Unknown	183 (20.3)	148 (23.7)	35 (12.7)
Tumor size (cm)			
≤ median (18.0)	451 (50.1)	303 (48.5)	148 (53.6)
>median	450 (49.9)	322 (51.5)	128 (46.4)
**Fertility-sparing surgery**			
Yes	411 (45.6)	306 (49.0)	105 (38.1)
No	490 (54.4)	319 (51.0)	171 (61.9)
**Scope Reg LN**			
1 to 3	79 (8.8)	53 (8.5)	26 (9.4)
4 or more	489 (54.3)	320 (51.2)	169 (61.2)
None/Other	333 (36.9)	252 (40.3)	81 (29.4)

G1, well differentiated.

G2, moderately differentiated.

G3, Grade 3, low differentiated or undifferentiated.

Scope Reg LN, Scope of Regional Lymph Node Surgery.

A total of eligible patients with stage I MOC were screened from the SEER database. Most of them were 31-45 years old (64.4%), white (74.8%), unmarried (45.3%), grade 1 (38.7%), and stage IA/IB (71.6%). A total of 490 (54.4%) patients underwent non-fertility sparing and 411(45.6%) patients underwent fertility-sparing surgery, 276(30.6%) patients received postoperative adjuvant chemotherapy.

### Determinants of chemotherapy

**Table 2** shows the distributions of patient characteristics according to chemotherapy treatment using univariate and multivariate logistic regression. Both univariate and multivariate logistic regression produced results that were comparable. According to a multivariate logistic regression study, individuals who were aged 31-45, with grade 2/3, stage IC, and no-fertility sparing surgery were more likely to receive adjuvant chemotherapy in real world. Older age was linked to greater odds of receiving chemotherapy in the multivariate logistic regression analysis (vs. 9–30 years old, OR:2.215, 95% CI: 1.443–3.401, P < 0.001). Patients with grades 2 and 3 had increased likelihood of receiving chemotherapy than those with grade 1 (OR: 3.712, 95% CI: 2.490-5.533, P < 0.001; and OR: 7.382, 95% CI: 4.054-13.443, P<0.001). Additionally, individuals with stage IC had higher odds of receiving chemotherapy than those with stage IA or IB (OR: 6.436, 95% CI: 4.515-9.175, P < 0.001).

**Table 2:** Logistic regression for associations between patient characteristics and chemotherapy.

Variable	Univariate		Multivariate	
	OR (95% CI)	p-value	OR (95% CI)	p-value
**Age (years)**				
9-30	1 (reference)		1 (reference)	
31-45	1.243 (0.928-1.667)	0.145	2.215 (1.443-3.401)	<0.001
**Race**				
Black	1 (reference)		1 (reference)	
White	0.594 (0.332-1.063)	0.080	0.522 (0.525-0.253)	0.078
Other/Unknown	0.684 (0.361-1.299)	0.246	0.520 (0.235-1.152)	0.107
**Marital status**				
Married	1 (reference)		1 (reference)	
Single (never married)	1.169 (0.861-1.586)	0.317	0.991 (0.669-1.469)	0.965
Other/Unknown	1.350 (0.863-2.114)	0.189	1.520 (0.907-2.547)	0.112
**CA125**				
Negative/normal	1 (reference)		1 (reference)	
Positive/elevated	1.727 (1.129-2.639)	0.012	1.530 (0.936-2.499)	0.090
Other/Unknown	0.980 (0.663-1.447)	0.917	0.971 (0.619-1.521)	0.896
**FIGO Stage**				
IA/IB	1 (reference)		1 (reference)	
IC	5.535 (4.041-7.581)	<0.001	6.436 (4.515-9.175)	<0.001
**Grade**				
1	1 (reference)		1 (reference)	
2	3.153 (2.223-4.474)	<0.001	3.712 (2.490-5.533)	<0.001
3	5.629 (3.308-9.579)	<0.001	7.382 (4.054-13.443)	<0.001
Unknown	0.960 (0.610-1.509)	0.859	1.292 (0.778-2.145)	0.321
**Tumor size (cm)**				
≤ median (18.0)	1 (reference)		1 (reference)	
>median	0.814 (0.613-1.081)	0.155	0.861 (0.614-1.208)	0.387
**Fertility-sparing surgery**				
Yes	1 (reference)		1 (reference)	
No	1.562 (1.170-2.086)	0.003	2.226 (1.490-3.327)	<0.001
**Scope Reg LN**				
1 to 3	1 (reference)		1 (reference)	
4 or more	1.526 (0.897-2.598)	0.119	1.180 (0.633-2.201)	0.602
None/Other	1.643 (1.203-2.245)	0.002	1.614 (1.114-2.338))	0.011

G1, well differentiated.

G2, moderately differentiated.

G3, Grade 3, low differentiated or undifferentiated.

Scope Reg LN, Scope of Regional Lymph Node Surgery.

### Predictors for survival

**Figure 2:**
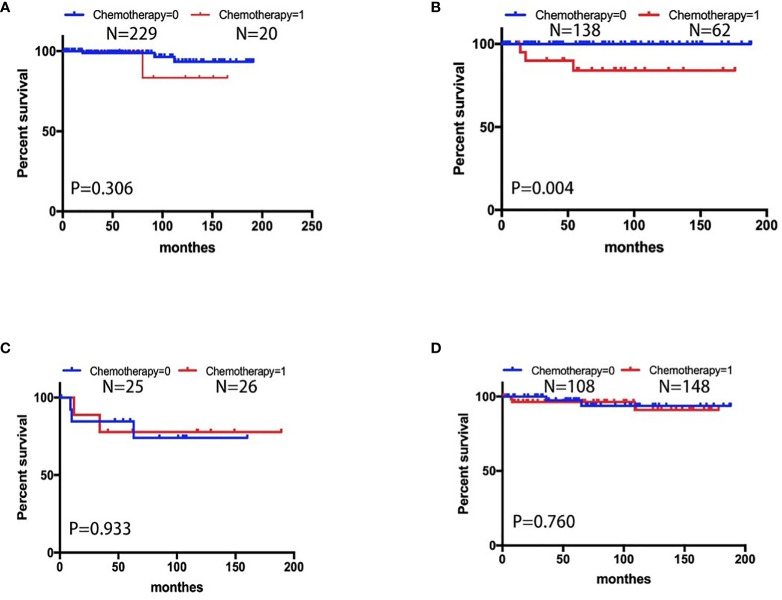
CSS curves stratified in stage I MOC by chemotherapy. **(A)** Stage IA/IB-grade 1, 10-year CSS rates of chemotherapy = 83.33%, non-chemotherapy = 93.39%); **(B)** stage IA/IB-grade 2, 10-year CSS rates of chemotherapy = 84%, non-chemotherapy = 100%; **(C)** stage IA/IB-grade 3, 10-year CSS rates of chemotherapy=77.778%, non-chemotherapy = 74.038%; **(D)** stage IC, 10-year CSS rates of chemotherapy = 90.947%, non-chemotherapy = 93.688% [0 = non-chemotherapy; 1 = chemotherapy].

We aimed to determine the impact of chemotherapy and non-chemotherapy on the special population with fertility preservation by screening the SEER database. A total of 411 eligible patients with stage I MOC who underwent fertility-sparing surgery were screened. Most of them were 9-30 years old (60.5%), white (73.7%), unmarried (58.6%) and stage IA/IB (72.1%). A total of 162 (39.4%) patients with grade 1, 128(31.1%) with grade 2, 34 (8.2%) with grade 3 and 105(25.6%) patients received postoperative adjuvant chemotherapy. First, we performed univariate analysis and found that chemotherapy was associated with survival as shown in **Table 3**. Then, according to the multivariate Cox regression model in **Table 4**, patients who received chemotherapy or who had grade 3 cancer had worse outcomes than other patients (HR: 2.905, 95% CI: 0.938-6.030, P=0.068 and HR: 4.750, 95% CI: 1.419-15.896, P=0.011). In addition, individuals who were older (HR: 0.845, 95% CI: 0.3432.079, P=0.713) had no survival difference compared to the former (**Table 4**). **Figure 2** displays CSS curves stratified by chemotherapy. Significant CSS differences were observed between the non-chemotherapy and chemotherapy groups in MOC when patients were stage IA/IB-grade 2 (P=0.004) (10-year CSS rates of chemotherapy=84%, non-chemotherapy = 100%), but not when they were stage IA/IB-grade 1(10-year CSS rates of chemotherapy=83.33%, non-chemotherapy 93.39%), stage IA/IB-grade 3 (10-year CSS rates of chemotherapy=77.778%, non-chemotherapy = 74.038%), or stage IC (10-year CSS rates of chemotherapy=90.947%, non-chemotherapy = 93.688%) (both P>0.05) (**Figure 2**).

**Table 3:** Univariate analysis of cancer-specific survival among stage I MOC patients with FSS.

Variable	Univariate		
	HR	(95% CI)	p-value
**Age (years)**			
9-30	1 (reference)		
31-45	1.072	0.449-2.556	0.876
**Race**			
Black	1 (reference)		
White	0.347	0.078-1.540	0.164
Other/Unknown	0.692	0.144-3.334	0.646
**Marital status**			
Married	1 (reference)		
Single (never married)	1.013	0.409-2.511	0.977
Other/Unknown	0.374	0.046-3.047	0.358
**CA125**			
Negative/normal	1 (reference)		
Positive/elevated	0.528	0.088-3.162	0.485
Other/Unknown	1.356	0.390-4.711	0.632
**FIGO Stage**			
IA/IB	1 (reference)		
IC	0.825	0.304-2.236	0.705
**Grade**			
1	1 (reference)		
2	1.340	0.388-4.631	0.643
3	6.286	1.994-19.824	0.002
Unknown	1.492	0.431-5.162	0.527
**Tumor size (cm)**			
≤ median (15.0)	1 (reference)		
>median	1.640	0.688-3.910	0.265
**Scope Reg LN**			
1 to 3	1 (reference)		
4 or more	0.561	0.071-4.429	0.584
other	1.383	0.583-3.283	0.462
**Chemotherapy**			
No	1 (reference)		
Yes	2.822	1.223-6.512	0.015

G1, well differentiated.

G2, moderately differentiated.

G3, Grade 3, low differentiated or undifferentiated.

Scope Reg LN, Scope of Regional Lymph Node Surgery.

**Table 4:** Multivariate analysis of cancer-specific survival among stage I MOC patients with FSS.

Variable	Multivariate		
	HR	(95% CI)	p-value
**Age (years)**			
9-30	1 (reference)		
31-45	0.845	0.343-2.079	0.713
**CA125**			
Negative/normal	1 (reference)		
Positive/elevated	1.067	0.300-3.797	1.067
Other/Unknown	0.358	0.058-2.210	0.358
**Grade**			
1	1 (reference)		
2	1.161	0.329-4.098	0.816
3	4.750	1.419-15.896	0.011
Unknown	1.418	0.4064.953	0.548
**Chemotherapy**			
No	1 (reference)		
Yes	2.905	0.938-6.030	0.068

G1, well differentiated.

G2, moderately differentiated.

G3, Grade 3, low differentiated or undifferentiated.

Scope Reg LN, Scope of Regional Lymph Node Surgery.

### Construction of a nomogram model of CSS

**Figure 3:**
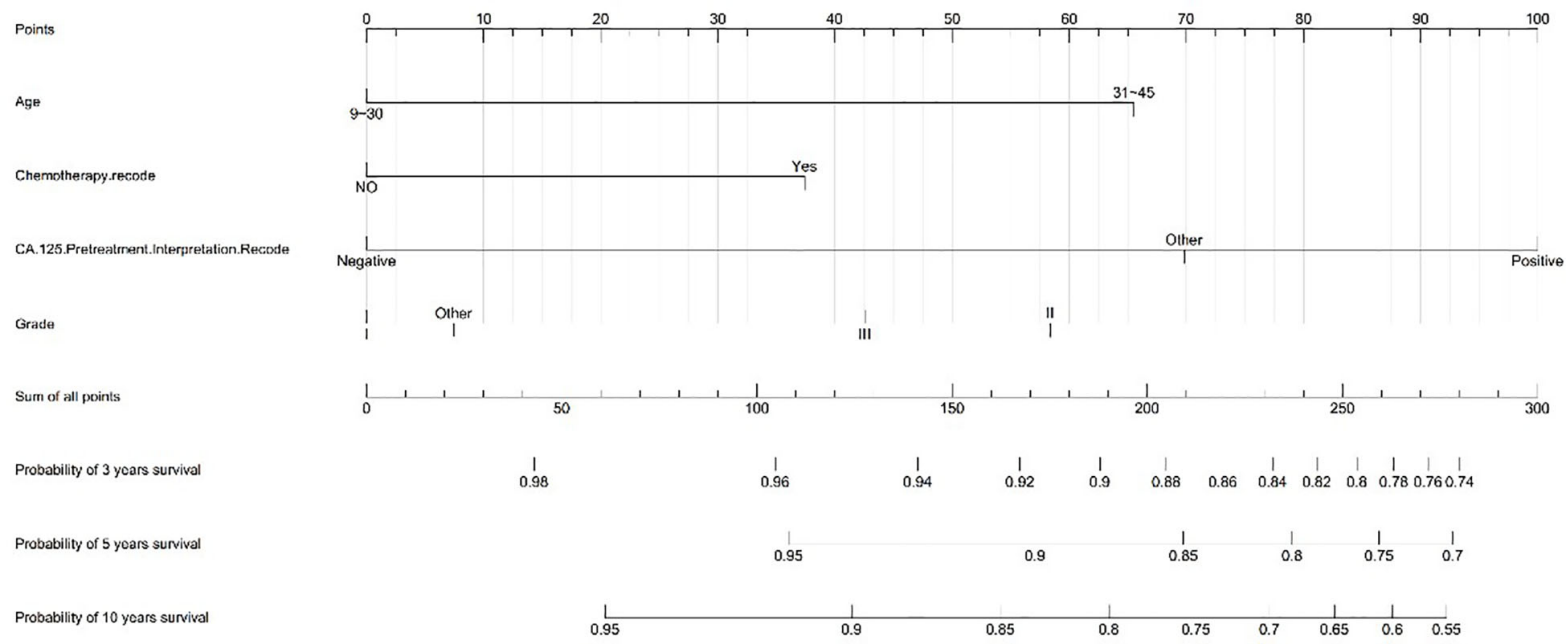
Nomograms to predict 3-, 5-, and 10-year CSS for stage I MOC.

We created a nomogram model of CSS using significant features among stage I MOC patients. The scores for each characteristic ranged from 0 to 100, and the aggregate of these scores, which ranged from 0 to 240, was also evaluated in accordance based on the 3-, 5-, and 10-year survival rates, which varied from 0.1% to 0.9%. (**Figure 3**).

### Calibration chart among patients

**Figure 4:**
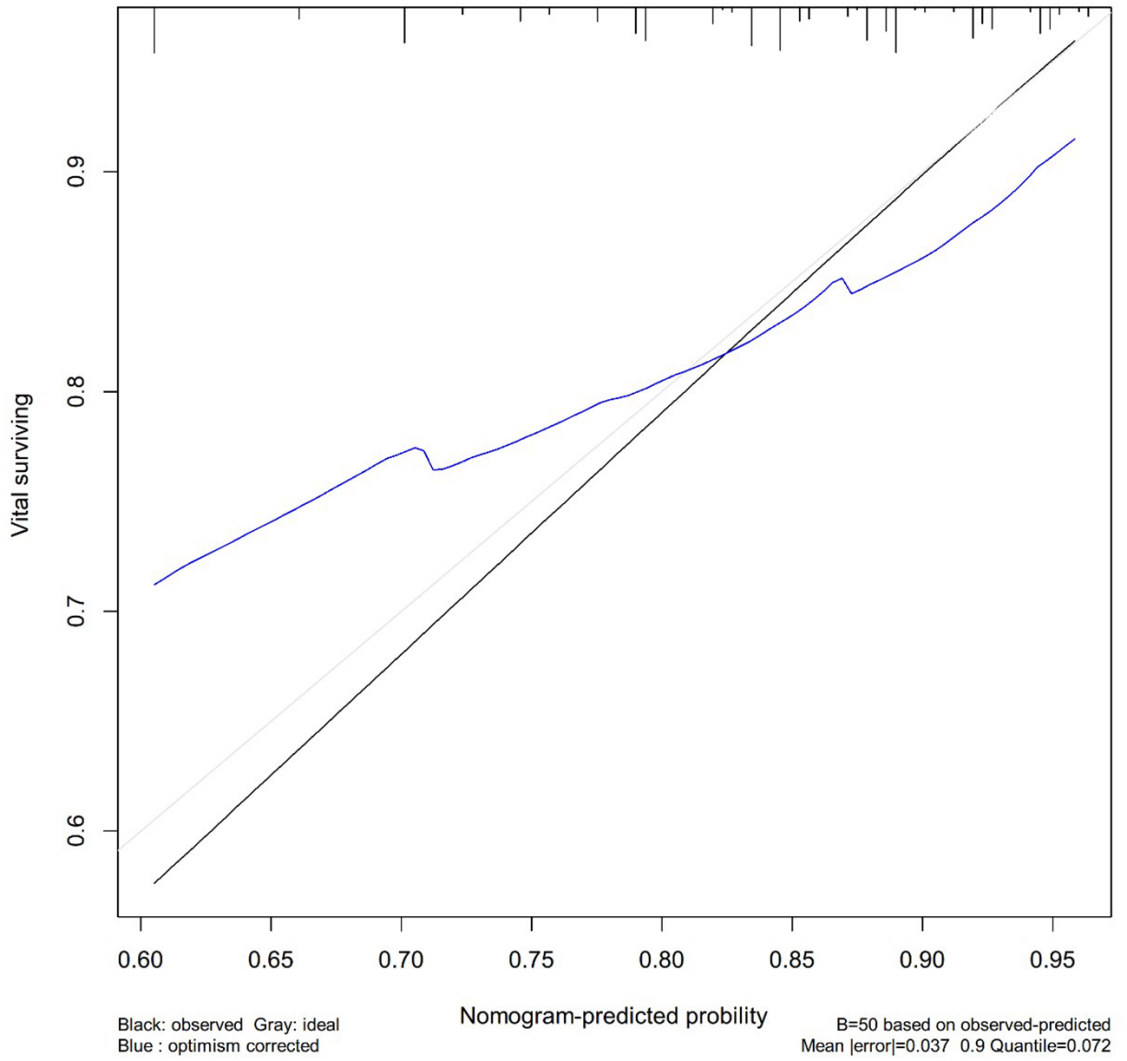
The calibration plot established for the nomogram among patients of stage I MOC.

The C-index for females with stage I MOC was 0.709. We created a calibration plot to show a positive prediction for 3-year and 5-year CSS among patients with fertility preservation to further assess the consistency of the nomogram (**Figure 4**).

## Discussion

MOC is a rare epithelial ovarian malignancy, accounting for approximately 3% (1). Because of low incidence, it was extremely difficult to conduct prospective clinical randomized controlled trials. However, by reviewing data in the SEER database, we studied the population of patients aged 31-45, with grade 3, stage IC, and non-fertility-sparing surgery being more likely to receive adjuvant chemotherapy in real world. With the development of assisted reproductive technology, the fertility-sparing surgery is no longer limited to the preservation of the uterus and ovary. We defined fertility-sparing surgery as uterine-sparing surgery for patients with stage I MOC within 45 years old in the study. Among them, for the special population of fertility preservation, grade 3 and chemotherapy were independent risk factors for prognosis, and the mortality risk of grade 3 patients with grade 1 increased by 4.7 times. Meanwhile chemotherapy increased the risk of death by nearly three times. In addition, adjuvant chemotherapy did not improve CSS when patients were in stage IA/IB-grade 1, stage IA/IB-grade 3, and IC stages; when patients were in stage IA/IB-grade 2, adjuvant chemotherapy reduced CSS rates. Then, we established a relatively reliable chemotherapy prediction nomogram model to guide clinicians to individualize treatment.

Chemotherapy is always used in epithelial ovarian cancer and is an independent risk factor affecting prognosis (20). Of them, the characteristics and biological behavior of MOC are quite different from common ovarian serous carcinoma. clinically, mucinous ovarian cancer has a young onset, with stage I accounting for up to 70%, and is not sensitive to chemotherapy. Early-stage patients have a good 5-year overall survival rate (8). In addition, some patients with early-stage MOC required fertility preservation. However, whether early-stage MOC patients benefit from systemic therapy is still unclear and especially for patients with stage IC, postoperative adjuvant chemotherapy remains controversial. The latest NCCN guidelines recommend postoperative chemotherapy or observation. Using case data from the SEER database, we performed multivariate logistic regression analysis to show that patients aged 31-45, with grade 3, stage IC, and non-fertility-sparing surgery were more likely to receive adjuvant chemotherapy in real world. Most of the results were consistent with those reported in previous studies (21). Our results showed that the 31- to 45-year-old age group was more likely to receive chemotherapy, in contrast to the previous study in which patients under 30 years of age were more likely to choose chemotherapy (21). Interestingly, we also found that patients who underwent fertility-sparing surgery were less likely to receive adjuvant chemotherapy than patients with non-fertility sparing surgery. The reason was that our study population was stage I patients with potential reproductive function within 45 years of age. Clinically, doctors may be concerned about the effects of chemotherapy on reproductive function in young female patients, thereby influencing chemotherapy choices. The effect of chemotherapy exposure on ovarian function in young ovarian cancer patients with fertility preservation remains unclear. Reviewing the previous study, most of the population was young women with non-epithelial ovarian cancer (22). Yang B et al. reported that 129 patients with non-epithelial ovarian cancer who received fertility-sparing surgery and adjuvant chemotherapy did not have ovarian failure. Of the 44 women who tried to conceive, 35 (79.5%) patients had 51 successful pregnancies, including 35 live births without birth defects. The investigators concluded that non-epithelial ovarian tumors have a satisfactory prognosis after fertility-sparing surgery and chemotherapy with little effect on fertility (22). Ceppi L et al. recently conducted an analysis of whether chemotherapy in patients with fertility-sparing surgery was associated with aamenorrhea, conception rate, pregnancy outcome and age at spontaneous menopause during and after treatment. The results showed that chemotherapy in non-epithelial ovarian cancer was associated with an increased risk of chemotherapy for non-epithelial ovarian cancer and was associated with increased risk of amenorrhea, post-treatment amenorrhea, and age at early spontaneous menopause during treatment while chemotherapy in epithelial ovarian cancer was not associated with either of these factors (23). Most studies concluded that chemotherapy had no significant effect on ovarian function in patients with fertility preservation. However, mucinous ovarian cancer patients are young at onset and not sensitive to chemotherapy, and there is a lack ofdirect research on the impact on ovarian function.

In clinical practice, poorly differentiated and stage IC MOC are more inclined to use adjuvant chemotherapy. However, for this rare and chemotherapy-insensitive disease, it is unclear whether patients benefit from chemotherapy, especially young patients with preserved fertility. Previous studies of chemotherapy for patients with epithelial ovarian cancer diagnosed at an early-stage typically covered all histopathological types, which contained a very low proportion of mucinous ovarian cancer. In a reanalysis of two prospective clinical studies of ICON1/ACTION, the 5-year overall survival rates between chemotherapy and no chemotherapy were 82% and 74% (HR=0.67, 95% CI=0.50-0.90; P=0.008). The 5-year recurrence-free survival rate in the adjuvant chemotherapy group was also better than that in the non-chemotherapy group (HR=0.6495%CI=0.50-0.82;P=0.001). Meanwhile, subgroup analyses revealed that the benefit of adjuvant chemotherapy appeared to be restricted to patients with incomplete staging, who were at a higher risk of having residual disease.However, the study also did not account for subgroup data of mucinous ovarian cancer patients because of small sample size (24). The results of previous retrospective studies have shown inconsistent conclusions about whether chemotherapy was beneficial to the survival of patients with early-stage MOC. A population-based retrospective study by Kumar A et al. was the first to demonstrate that chemoradiotherapy improves survival rates in patients with stage I or stage II ovarian mucinous carcinoma. However, adjuvant therapy was not recommended for patients with stage IA grades I and II (25). Two database studies focusing on stage I MOC patients were recently published. Less than 60% of stage IC MOC patients received postoperative adjuvant chemotherapy, and the results showed that chemotherapy did not improve survival outcomes. Adjuvant chemotherapy did not benefit survival outcomes regardless of patient age, tumor size, stage I substage, or degree of differentiation (26, 27). Using a clinical nomogram and a chemotherapy prediction scoring methodology, the authors assessed the long-term survival benefit of adjuvant chemotherapy in patients with high-risk stage I mucinous ovarian cancer. The 10-year overall survival rate for those who did not receive chemotherapy was 88% and 84% for those who received chemotherapy. Adjuvant chemotherapy patients experienced comparable rates of survival and mortality risks (HR=0.80, 95%CI=0.56-1.15, P=0.23). However, treatment increased 10-year overall survival by 23% (74% vs 51%) in high-risk patients (n=405). The patients who did not receiveadjuvant chemotherapy in the high-risk group had a 58% higher mortality risk (95% CI 1.05-2.38, P=0.03) (28). With the development of assisted reproductive technology, the fertility-sparing surgery is no longer limited to the preservation of the uterus and ovary. We defined the fertility-sparing surgery as uterine-sparing surgery for patients with stage I MOC within 45 years old in the study. Grade 3 was also an independent risk factor for prognosis. This shows that regardless of whether the uterus is preserved and the stage I substaging, grade 3 predicts poor prognosis. More interestingly, for this particular group, choosing chemotherapy increased the risk of death. Data from previous studies have also found a trend toward lower CSS rates for patients with early-stage mucinous ovarian cancer who received chemotherapy, but there was no statistically significant difference (26, 27). For the special population of fertility preservation, chemotherapy brought a significant risk of death, which should be carefully selected in clinical practice. The reason for its increased risk of death may be related to chemotherapy-related toxicity and side effects, and the specific reasons need to be further studied.

In addition, we developed a prognostic prediction nomogram model for stage I fertility-sparing young ovarian mucinous carcinoma patients. The nomogram shows excellent calibration results and C-index. The ovarian cancer nomogram focuses on prognostic impact rather than providing guidelines for the use of adjuvant chemotherapy, especially for rare tumors (29). Our study focused on the 10-year overall survival rate, which has very important clinical value as an ovarian tumor model with a better prognosis. However, like all larger retrospective database studies, this study is limited by incomplete information. Chemotherapy regimens and number of cycles are lacking in the SEER database, and mucinous carcinoma patients may receive gastrointestinal chemotherapy regimens, although this effect might be less pronounced on outcomes in the first stage (30). The inability to obtain restaging in IC stage might also have a certain impact on the results, such as whether the intraoperative mass was ruptured, the ascites cytology was positive, etc. (31). Pathological diagnosis of mucinous ovarian carcinomas including borderline mucinous tumors, and well-differentiated tumors was often challenging. Pathological invasive patterns such as invasive and expansive growth may also represent different prognosis, but these data are not available (32). Due to the low incidence of mucinous ovarian cancer and the lower sample size of patients with fertility preservation, internal and external data model validation has not been performed, and more clinical information and samples should be used in future research to increase the reliability of the model.

In conclusion, for stage I MOC patients aged 31-45, grade 3, stage IC, and non-fertility-sparing surgery were more likely to receive adjuvant chemotherapy in real world. However, for the special population of fertility-sparing patients, the choice of chemotherapy may increase the risk of death, and it should be carefully selected in clinical practice. According to FIGO staging and classification, when patients were stage IA/IB-grade 2, adjuvant chemotherapy reduced CSS rates.

## Data availability statement

Publicly available datasets were analyzed in this study. This data can be found here: https://seer.cancer.gov/data-software/.

## Ethics statement

The informed consent was not required in this study, because personal identifying was not included in the SEER database.

## Author contributions

XL and RL contributed equally to this work. Study concept and design: DZ. Data acquisition: XL. Data analysis and interpretation: RL. Software: XL and RL. Y-mH. Critical revision: LY and YT. All authors contributed to the article and approved the submitted version.

## Funding

Beijing Kanghua Foundation for the Development of Traditional Chinese and Western Medicine “Le foundation” (KH-2020-LJJ-044, Xingtao Long).

## Conflict of interest

The authors declare that the research was conducted in the absence of any commercial or financial relationships that could be construed as a potential conflict of interest.

## Publisher’s note

All claims expressed in this article are solely those of the authors and do not necessarily represent those of their affiliated organizations, or those of the publisher, the editors and the reviewers. Any product that may be evaluated in this article, or claim that may be made by its manufacturer, is not guaranteed or endorsed by the publisher.
